# Risk Factors Associated with Dengue Transmission and Spatial Distribution of High Seroprevalence in Schoolchildren from the Urban Area of Medellin, Colombia

**DOI:** 10.1155/2018/2308095

**Published:** 2018-09-02

**Authors:** Leidy Diana Piedrahita, Ivony Y. Agudelo Salas, Katherine Marin, Andrea I. Trujillo, Jorge E. Osorio, Sair Orieta Arboleda-Sanchez, Berta Nelly Restrepo

**Affiliations:** ^1^Instituto Colombiano de Medicina Tropical, Universidad CES, Carrera 43 No. 52 Sur–99, Sabaneta, Antioquia 055450, Colombia; ^2^Department of Pathobiological Sciences, University of Wisconsin-Madison, 1656 Linden Drive, Madison, WI 53706, USA; ^3^Grupo de Biología y Control de Enfermedades Infecciosas -BCEI, Universidad de Antioquia, Calle 70 No. 52-21, Medellín, Antioquia 050010, Colombia

## Abstract

Dengue fever is an increasing health problem in tropical and subtropical regions. During 2010 in Medellin, the younger population presented a particularly high dengue incidence rate. This study estimated dengue virus (DENV) transmission in schoolchildren (aged 5–19 years) in Medellin from 2010 to 2012. A longitudinal serological survey (IgG) and spatial analysis were conducted to determine the distribution of DENV seroprevalence. A total of 4,385 schoolchildren participated for at least one year. Dengue seroprevalence significantly increased during the studied period (53.8% to 64.6%; *p* < 0.001). A significantly higher seroconversion rate was observed in 2010-2011 (16.8%) compared to 2011-2012 (7.8%). Multivariate regression analysis showed that the main factor associated with the seroprevalence was the aging. Furthermore, in 2010, patients with high socioeconomic status presented a lower risk. Predominant multitypic and DENV4 monotypic antibody responses were demonstrated. Geostatistical analysis evidenced a temporal clustering distribution of DENV seroprevalence in 2010. Population density and *Ae. aegypti* House Index were significantly correlated with the observed pattern. This study revealed high DENV transmission in schoolchildren determined as “*sentinel population*.” High DENV risk was found in districts with combined poorly socioeconomic conditions and densest human and mosquito populations. These findings may allow to target population for effective prevention and vaccination campaigns.

## 1. Introduction

Dengue fever is an acute disease caused by dengue virus (DENV), an arbovirus that belongs to the Flaviviridae family, *Flavivirus* genus. DENV has four related antigenic serotypes (DENV1, DENV2, DENV3, and DENV4). The virus is transmitted by the bite of infected mosquitos from the *Aedes* genus, mainly *Aedes aegypti* and *Aedes albopictus* [1]. DENV is the most prevalent arbovirus in humans, and it is considered a major public health problem in the tropical and subtropical regions [2]. Dengue fever has a vast clinical spectrum from asymptomatic, subclinical, undifferentiated febrile syndrome to severe disease [3]. In 2009, the World Health Organization (WHO) proposed to categorize the disease into dengue and severe dengue [2]. Severe dengue can be caused by any serotype [4] and is characterized by plasma leakage, bleeding, and/or organ injuries [5]. It is estimated that about 390 millions of DENV infections occur annually, amongst which only 96 million are symptomatic infections [6]. During the decade 2000–2010, Latin America and the Caribbean region reported particularly high numbers of dengue cases and additional transmission of all four DENV serotypes. Colombia, Brazil, and Venezuela were the countries with the largest outbreaks [7]. Colombia registered 147,257 cases of dengue, 9,755 cases of severe dengue, and 217 deaths; the incidence rate was 345.4 cases × 100,000 inhabitants, and the dengue lethality was 2.28% [8]. All serotypes were reported, mostly DENV2 (44%) and DENV1 (41%). The Colombian National Institute of Health (Instituto Nacional de Salud (INS)) declared that dengue infection in Colombia followed an endemic-epidemic trend and that the risk of transmission covered 85% of its territory. The disease expanded and intensified in the whole country with cyclic outbreaks every two or three years [8, 9]. The average ages of dengue and severe dengue during the 2010 outbreak were twenty-six and twenty-two years, respectively [8]. The local health authorities of Medellin (Secretaria de Salud de Medellin), the capital city of Antioquia Department, reported during the same year that the group with the highest incidence of dengue was aged 15–19 years and that 28% of the cases occurred in scholar population. Moreover, 117 severe dengue cases and 14 deaths were reported. Dengue lethality was about 12%, and global mortality was 0.6 cases × 100,000 inhabitants [9]. Dengue burden in Medellin is underestimated mostly because incidence and mortality are calculated based on the symptomatic cases reported to local authorities. However, it is well known that those reports represent only a fraction of the dengue transmission mostly because most of the cases are subclinical or asymptomatic infections.

Several countries have performed studies of seroprevalence and seroconversion in scholar population, under the assumption that the findings should reflect dengue transmission in entire children and young populations [10–14]. However, little is known about the epidemiologic characteristics of DENV prevalence in the young populations. The aim of the present study was to estimate DENV transmission in schoolchildren population (aged 5–19 years) in Medellin, Colombia. The specific aims were (i) to determine sociodemographic factors associated with dengue seroprevalence (IgG antibodies) and to estimate the dengue seroconversion rate, (ii) to characterize the serotype of antibody responses in a random selection of serum samples, and (iii) to describe the role of population density and entomological indexes associated with the spatial distribution of DENV seroprevalence.

## 2. Methods

### 2.1. Study Setting

**Figure 1:**
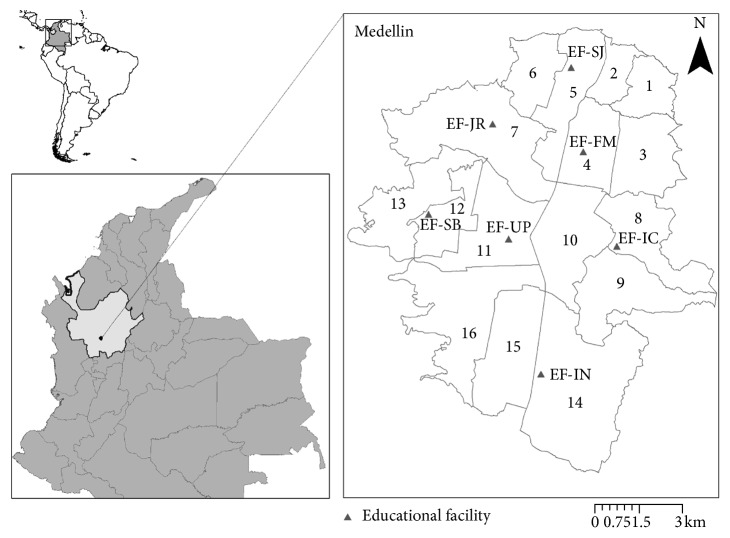
Map showing the location of the study sites (EFs) in Medellin, located in Antioquia Department, Colombia. On the right, the urban district boundaries of Medellin with its 16 urban districts (1-Popular, 2-Santa Cruz, 3-Manrique, 4-Aranjuez, 5-Castilla, 6-Doce de Octubre, 7-Robledo, 8-Villa Hermosa, 9-Buenos Aires, 10-La Candelaria, 11-Laureles-Estadio, 12-América, 13-San Javier, 14-Poblado, 15-Guayabal, and 16-Belén) and the spatial location of the seven educational facilities (triangles) are given.

Medellin is the capital city of Antioquia Department in Colombia. It has an area of 382 km^2^ and is located at 6°15′07″N, 75°33′49″W and at an altitude of 1,500 m.a.s.l. Its climate is considered tropical-subtropical, with an annual average temperature of 22°C and an average annual rainfall of 1,752 mm. Its territory is divided into sixteen urban districts (Figure 1) and five rural districts [15]. Medellin is one of the largest cities of Colombia, with an estimated population of 2,343,049 inhabitants in 2010 [16]. Amongst them, 509,009 inhabitants belong to the 5- to 19-year-old group [17]. There are about 703 educational facilities (EFs), and 397 of those are private.

### 2.2. Study Design

We conducted a longitudinal serological survey from May 2010 to May 2012, in schoolchildren population aged 5 to 19 years (determined as “*sentinel population*”) of seven EFs from Medellin.

### 2.3. Study Population

Inclusion criteria were schoolchildren enrolled at any educational stage, signature on an informed consent form by parents or legal guardians, and signature on an informed scholar assent form by schoolchildren when they were over 8 and less than 17 years old.

The study population was constituted by 1,983 schoolchildren from three EFs that were part of a longitudinal study previously published during 2010 [18]. In addition to these three EFs, other four EFs participated during 2011 with 848 schoolchildren. In 2012, only the last four EFs participated.

**Figure 2:**
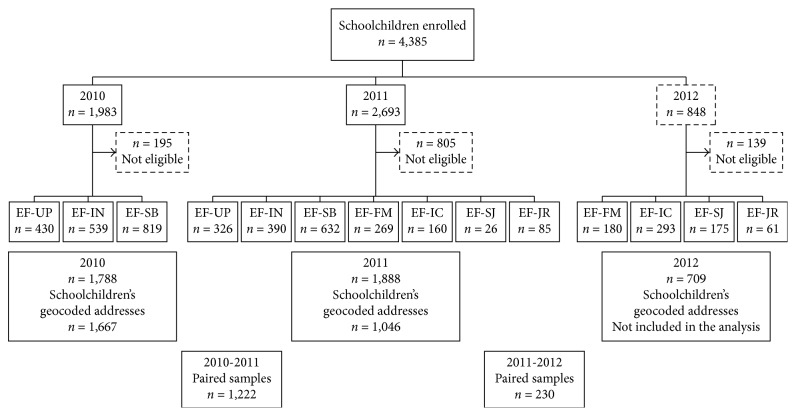
Flow chart of enrolled participants per educational facility (EF) and study year.

The sample size was calculated using Epi Info v6.0 with the following parameters: a universe of 339,229 schoolchildren for public EFs and of 70,208 schoolchildren for private EFs, 5% sampling error and 95% confidence interval (95% CI), and 50% of dengue seroprevalence, and then 10% was added in case of loss. The resulting total sample size required was 844 schoolchildren. The EFs were named as follows: EF-UP located in the Laureles-Estadio district, EF-SB located in the América district, EF-IN located in the Poblado district, EF-FM located in the Aranjuez district, EF-IC located in the Villa Hermosa district, EF-SJ located in the Castilla district, and EF-JR located in the Robledo district (see Figure 1 for spatial localization of each EF). Some participants (*n*=195, *n*=805, and *n*=139 in study years 2010, 2011, and 2012, respectively) were excluded from the analysis due to incomplete data collection or because their home addresses were georeferenced outside of Medellin. Overall, 1,667 and 1,046 participants were georeferenced and located in Medellin in 2010 and 2011, respectively. Paired serum samples were collected from 1,452 participants (*n*=1,222 in 2010-2011 and *n*=230 in 2011-2012) (Figure 2).

### 2.4. Data Collection

Two annual serological surveys were made during the study period at the EF sites. At these occasions, blood samples (3–5 ml) were obtained from scholar participants. The serum samples were tested for IgG antibodies against DENV using the enzyme-linked immunosorbent assay (ELISA) and the enzyme-linked immunospot and microneutralization tests (ELISPOT-MNT).

A survey form was sent to parents or legal guardians, and they were inquired about sociodemographic characteristics and participant's history of dengue infection.

Geographical coordinates of each EF using a GPS (global positioning system) device (Garmin Co) with 3 m accuracy and participant's address of the residence were obtained. The digital maps with administrative boundaries of the urban area of Medellin represented as polygons were obtained from the website of the local department of administrative planning [15]. Data population to calculate population density (inhabitants/km^2^) of each district was obtained from published reports of the local statistic department [17]. Additionally, entomological indices (House Index (HI), Container Index (CI), Breteau Index (BI), and adult population density) were obtained from the local health authorities (Secretaria de Salud, vigilancia domiciliaria, municipio de Medellin). Spatial analyses were conducted using the presence of IgG antibodies against DENV as the dependent variable.

### 2.5. Antibodies Detection

#### 2.5.1. DENV IgG Antibodies

In-house dengue IgG indirect ELISA was standardized to determine IgG antibodies in serum samples from participants [19, 20].

Enzyme-linked immunospot and microneutralization tests (ELISPOT-MNT) [21] were performed in 278 serum samples following a standardized previously published protocol [22]. The ELISPOT-MNT_50_ titer was determined as the serum dilution that reduces the number of foci or plaques by at least 50% when compared with wells with virus control.

### 2.6. Study Definitions

#### 2.6.1. Past Dengue Virus Infection

It was considered that the participants with detectable levels of DENV IgG antibodies evidenced by in-house ELISA had a past dengue virus infection.

#### 2.6.2. Seroconversion

It was considered that the absence of DENV IgG antibodies in the first sample taken from the participant followed by a positive detection in the second year of the survey evidenced DENV seroconversion.

#### 2.6.3. Monotypic or Multitypic Immune Responses

Monotypic or multitypic immune responses were defined, respectively, as positive responses against single or multiples serotypes of DENV, as determined by ELISPOT-MNT [23].

### 2.7. Data Analysis

Statistical analysis was conducted using the Statistical Package for the Social Sciences (SPSS®, version 21; SPSS Inc., Chicago, IL, USA). The database was generated in Microsoft Excel (Microsoft Corp., Redmond, WA, USA). Median and interquartile range were calculated for quantitative variables. Absolute and relative frequencies were calculated for qualitative variables. Bivariate analysis was used to test the hypotheses of association between qualitative variables using Pearson's chi-squared test (*X*^2^) and chi-squared test for the trend with the corresponding number of degrees of freedom (df). The Mann–Whitney *U* test was used to determine the difference between distributions of quantitative variables. The probability distribution of quantity variables was estimated using the Kolmogorov–Smirnov test. Univariate and multivariate logistic regression was performed to determine sociodemographic variables related to DENV IgG seroprevalence. The entry method was used to estimate model fitness. Adjusted odds ratio (OR, with 95% CI) of DENV IgG seroprevalence was calculated as an “explanatory variable” and was included in the model (gender, age group, ethnicity, and socioeconomic status). Statistical significance was considered for a *p* value lower than 0.05 (*p* < 0.05).

### 2.8. Spatial Analysis

Mapping of DENV IgG prevalence and spatial analysis were conducted in the ArcMap tool of the ArcGIS v10.4.1 software (Environmental Systems Research Institute (ESRI), Redlands, California, US). Two vector layers were used for analysis: one with point data of each participant's geocoded address associated with IgG results in 2010 and 2011 and the other with polygon data of the urban district boundaries of Medellin. Then, layer maps were associated using the spatial join tool implemented in ArcGIS. The inverse distance weighted (IDW) interpolation was used to produce a prediction of DENV IgG seroprevalence to any unmeasured location in the urban area of Medellin. Spatial autocorrelation of positive IgG subjects was evaluated with the Global Moran's I statistic. A positive value indicated a tendency toward clustering, while a negative value indicated a tendency toward dispersion [24]. Then, an exploratory regression analysis was performed to evaluate the spatial relationship of the observed DENV IgG seroprevalence patterns. Linear regression with a combination of possible explanatory variables (population density and indicators of *Aedes aegypti* House Index (HI), Breteau Index (BI), and Container Index (CI)) was performed to generate a prediction that best explains DENV IgG seroprevalence in each district of Medellin.

### 2.9. Ethics Review

This study was approved by the Ethical Review Committee of Instituto Colombiano de Medicina Tropical (ICMT) and Universidad CES.

## 3. Results

### 3.1. Sociodemographic Characteristics

Participants' median age was 11 years (IQR 9–13), 12 years (IQR 10–14), and 11 years (IQR 9–13) and women percentage was 51.5%, 52.8%, and 52.9% in 2010, 2011, and 2012, respectively. Most of the participants self-identified themselves as mestizo (96.1%), and the most frequent age group was 10–14 years (56.5%). All socioeconomic statuses (SESs) were represented, but low and medium SESs prevailed. Through 2012, there were not scholars belonging to a high SES (Table 1).

### 3.2. DENV Transmission in Schoolchildren Population Aged Five to Nineteen Years during 2010–2012

#### 3.2.1. Dengue IgG Seroprevalence

The detection of IgG antibodies is a serologic indication of past DENV infection. The frequency of these antibodies shows a statistically significant increase during the studied years. Dengue IgG frequency was 53.8% (95% CI = 51.5–56.1) in 2010, 58.5% (95% CI = 56.2–60.7) in 2011, and 64.6% (95% CI = 61.0–68.2) in 2012, (*X*^2^=25.137; *p*=0.004). In contrast, only 5.1% (91/1,788), 4.9% (92/1,888), and 4.1% (29/709) of participants self-reported past DENV infection each year, respectively.

#### 3.2.2. Dengue Seroprevalence and Age

**Figure 3:**
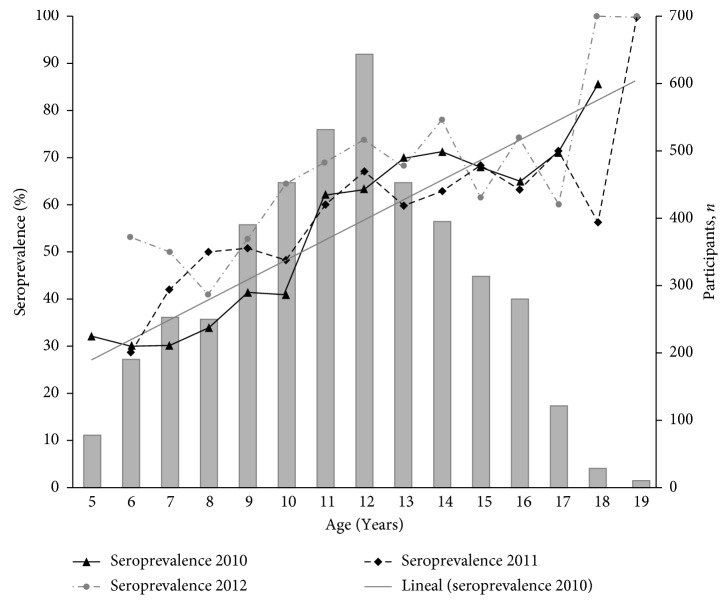
Seroprevalence of IgG antibodies against dengue virus related to patient's age in the studied scholar population in 2010–2012 from Medellin.

A significant correlation between the age of the patients and the prevalence of IgG antibodies was observed during the three studied years (*X*^2^=152.746; *p* < 0.001). DENV seroprevalence found in this scholar population was 30% for 5-year-old patients and 100% for 18-year-old patients (Figure 3).

#### 3.2.3. Sociodemographic Risk Factors Associated with DENV Seroprevalence in Schoolchildren Population

Univariate logistic regression analysis revealed that the risk of dengue prevalence increased with study progress (Table 2). Both univariate and multivariate logistic regression analysis (Tables 2 and 3) revealed that the main factor associated with DENV prevalence was the increase in scholar's age. Also, socioeconomic status appeared as a significant risk factor, with high-SES patients showing lower risk compared to low-SES patients in 2010 (OR = 0.59; 95% CI = 0.39–0.87; *X*^2^=20.198; *p* < 0.001). In 2012, higher risk to DENV exposition was observed in women (OR = 1.38; 95% CI = 1.01–1.89).

### 3.3. Characterization of DENV Antibody Responses in Schoolchildren Population

The characterization of antibody responses to dengue virus was performed using ELISPOT-MNT_50_ in a random selection of 278 serum samples. The median age of participants evaluated was 11 years (IQR = 8–14). During the study period, a multitypic response was mainly found (72.3%; 201/278). When monotypic, the response was mostly directed against DENV4 (Table 4).

#### 3.3.1. DENV IgG Seroconversion during the 2010-2011 and 2011-2012 Periods

**Figure 4:**
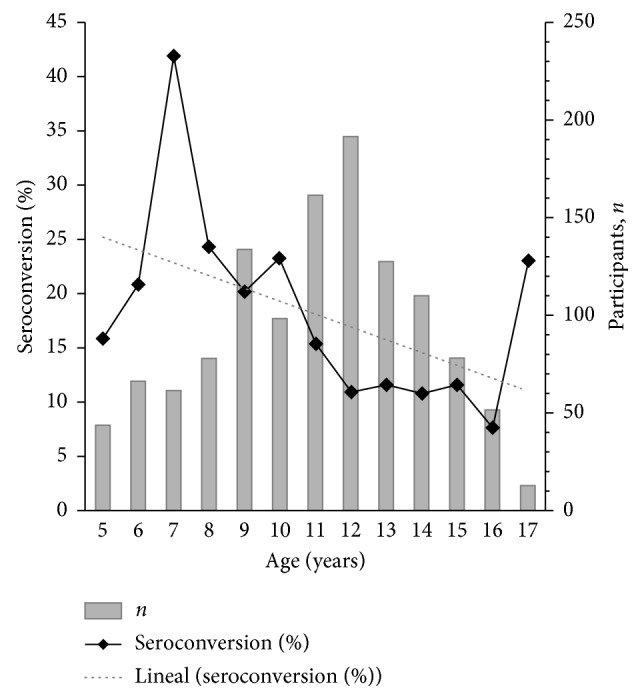
The seroconversion of IgG antibodies against DENV related to patient's age in the studied schoolchildren population from Medellin through 2010-2011.

Paired samples were collected from a total of 1,452 participants: 1,222 samples out of 1,788 (68.3%) during the 2010-2011 period and 230 out of 1,888 (12.2%) during the 2011-2012 period (Figure 2). Seroconversion was significantly higher during the 2010-2011 period (16.8%; 205/1,222) as compared to the 2011-2012 period (7.8%; 18/230) (*X*^2^=15.128; *p*=0.002). Additionally, during the 2010-2011 period, the seroconversion rate was higher in patients aged 5–9 years (24.2%, with a 41.9% peak for 7-year-old patients). A significant relation between age and IgG antibodies seroconversion was observed during this period (*X*^2^=24,519; *p* < 0.001) (Figure 4).

### 3.4. Mapping and Geostatistical Analysis of DENV IgG Seroprevalence Distribution in Medellin during 2010 and 2011

**Figure 5:**
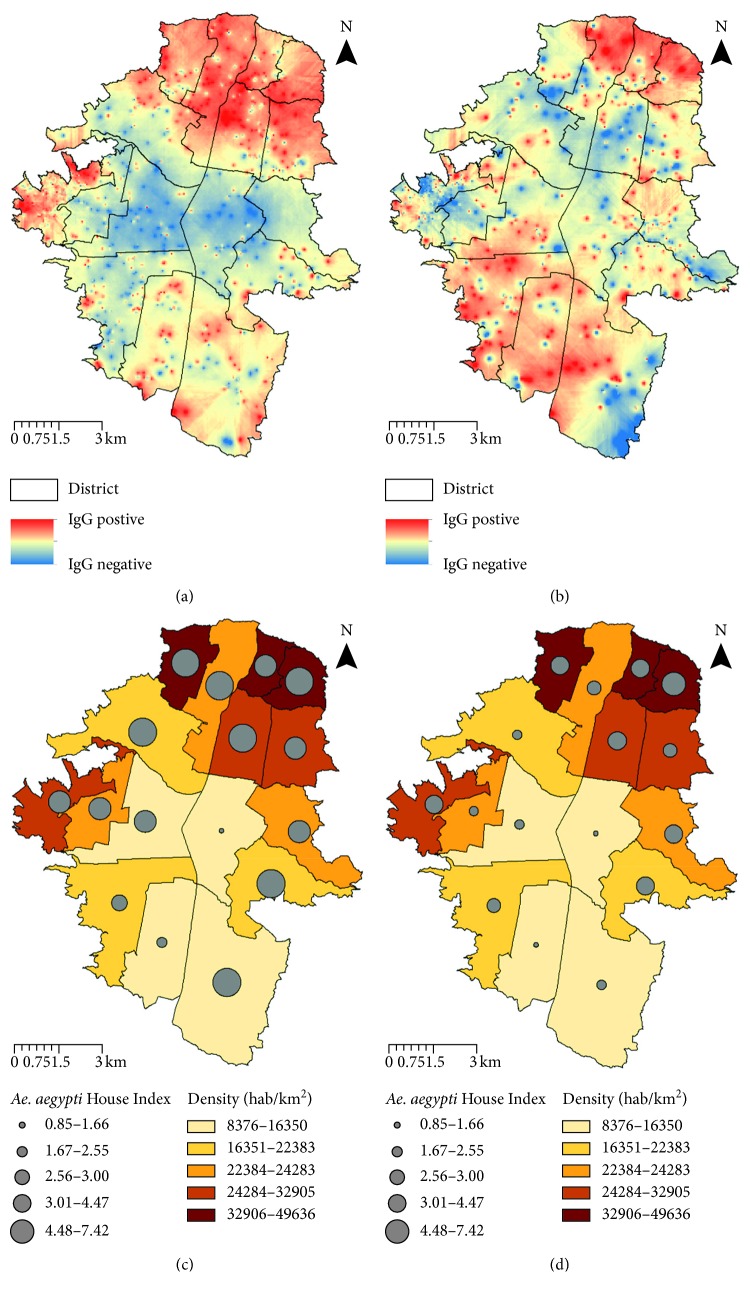
Spatial DENV IgG seroprevalence analysis in Medellin 2010 and 2011. Inverse distance weighting (IDW) interpolation using a set of points of schoolchildren DENV IgG seroprevalence results during 2010 (a) and 2011 (b). Superimposed layers: one with the graduated colors layer represents density and the second with the graduated symbols layer represents *Ae. aegypti* House Index (HI) in each district of the urban area of Medellin during 2010 (c) and 2011 (d).

In the present study, 1,667 and 1,046 participants with their parents' consent were georeferenced during 2010 and 2011, respectively (Figure 2). It represents 0.33% (1,667/509,009) and 0.21% (1,046/501,862) of the total population of Medellin aged 5 to 19 years each year, respectively [17]. The participants were located in all districts, with San Javier (13) district being overrepresented (46.0% (767/1,667) and 49.2% (515/1,046) of all participants in 2010 and 2011, respectively). The predicted DENV IgG seroprevalence in the urban area of Medellin displayed a temporal clustering distribution during 2010 (Figure 5(a)). Thus, Global Moran's I test was significant with a positive spatial autocorrelation of 0.04 (*z*-score = 2.065; *p*=0.038). In contrast, during 2011, DENV IgG seroprevalence distribution was dispersed (Figure 5(b)). Accordingly, Global Moran's I score was 0.002 (*z*-score: 0.01; *p*=0.922).

The diagnostic of the exploratory regression model indicated redundancy among the explanatory variables, specifically the entomological indices, as displayed by a large variance inflation factor (VIF > 7.5). At that point, the model was restarted using these indicators separately. Interestingly, spatial variability of the population density (adjusted *R*^2^=0.38; *p* < 0.01) and *Ae. aegypti* House Index (adjusted *R*^2^=0.28; *p* < 0.05) displayed a significant positive correlation with the observed DENV IgG seroprevalence. During 2010, this model explained 43% of the observed pattern over the urban area of Medellin (adjusted *R*^2^=0.43; *p* < 0.05). During 2011, these variables had no significant impact over the spatial pattern observed. ArcMap was employed to generate a superimposed surface of density population and vector House Index (HI) over districts of Medellin (Figures 5(c) and 5(d)).

## 4. Discussion

DENV infection is a complex, cyclic, and time-varying disease [25]. In the present study, using a representative sample of schoolchildren population from districts of the urban area of Medellin, we combined serologic and sociodemographic data and entomologic and geospatial information to better understand DENV transmission patterns. This study is one of the few studies in the country [26] to describe the hidden scale of DENV infection, particularly during 2010, a year with the largest outbreak reported in the region over the last decade. The first principal finding of this study is that dengue IgG seroprevalence increased significantly along with the study progress in schoolchildren population. Multivariate regression results showed that the main factor associated with dengue IgG seroprevalence was the age of the schoolchildren since, at the third year of the study (2012), most of the of participants aged 18 years have been exposed to the virus. Furthermore, during the 2010-2011 period, significant seroconversion in younger population was observed. The seroconversion rate was higher in the 5- to 9-year-old subpopulation with a peak at seven years. High and age-specific DENV IgG seroprevalence is consistent with long-term endemic circulation of DENV [27], and it is in accordance with a recent community-based survey carried out in the Santa Cruz district of Medellin [26] after the 2010 outbreak. Similar results in younger population have been reported in countries like Nicaragua [10, 28], Dominican Republic [29], Southern Vietnam [14], and Venezuela [30].

Another finding of this study is the remarkably higher risk to dengue of woman population (especially observed in 2012). This is in accordance with other studies in Latin American countries, like Mexico [31]. In contrast, southern Asian countries reported that men had twice the risk to DENV exposition than women [31]. A specific attention should be paid to this woman population, considering that DENV infection in pregnant women may result in maternal morbidity/mortality and that the passive transfer of maternal IgG antibodies against DENV has been associated with fetal complications and severe infection in infants [32]. Our study also demonstrates the impact of socioeconomy, population density, and entomological indexes on the distribution of DENV. Multivariate logistic regression showed that participants with lower socioeconomic status had higher risk to DENV exposition in 2010. Furthermore, geostatistical regression analysis proved that, during the same year, DENV IgG seroprevalence was clustered and 40% of the pattern observed arised out of districts with crowded population density and higher *Ae. aegypti* House Index. Those findings evidence that, through 2010, the combination of poorly and crowded socioeconomic living conditions and densest human and mosquito populations was associated with a higher DENV transmission risk. As reported by other authors, those factors are determinants for the permanent DENV transmission [30]. The House Index (HI), Container Index (CI), and Breteau Index (BI) are recommended by PAHO (Pan American Health Organization) for monitoring vector abundance and targeting and evaluating vector control interventions. There is no consensus in the literature about the threshold of *Ae. aegypti* density measures associated with the increased risk of human DENV infection [25, 33]. In our study, only HI was significantly associated with DENV infection. The HI range is considered between low and middle transmission risks of DENV by *Ae. aegypti* based on PAHO entomological surveillance guidelines [34]. Several studies claimed that vector densities fail to describe current DENV infection risk [25, 35]. These discrepancies suggest the inadequacy of the current indices in dengue transmission, probably because they were initially developed for the evaluation of yellow fever transmission risk [36]. Additionally, human movements depend on social and economic factors and also govern DENV transmission through time and space [37, 38].These movements determine the introduction of DENV into *Ae. aegypti* population and the presence of susceptible individuals that mosquitoes infect, perpetuating new rounds of transmission and appearance of outbreaks [25]. Therefore, many gaps are still present in our knowledge model of the relationships between vector density, vector competence, human susceptibility, and severity of DENV infection [35]. Another important finding of this study is the small percentage of participants that self-reported history of DENV infection. A recent study evidenced the importance of subjects with natural DENV infections without clinical symptoms, as it revealed that this population was more infectious to mosquitoes than the clinically symptomatic patients [37]. The authors emphasized how this population could contribute to DENV-persistent circulation during interepidemic periods by efficiently infecting mosquito vectors. Additionally, it is described that some serotypes (such as DENV3 [39] and DENV2 [28]) produce more severe symptoms than the other serotypes. Thus, after an outbreak period, the multitypic cross-reactive immune response could be able to reduce dengue disease severity [31]. However, population exposed to new serotypes may experience enhanced immune response that results in clinically symptomatic cases. The 2010 outbreak resulted in an exceptional cocirculation of all four DENV serotypes in Colombia. Consistently, results of the ELISPOT-MNT_50_ showed a predominant multitypic response and a monotypic antibody response principally against DENV4. Even though, the higher frequency of serotypes isolated from symptomatic reported cases in the country was DENV2 and DENV1 [8]. Studies described that circulation of DENV4 serotype decreased due to cross-serotype responses [40–42] and may also affect clinical severity.

## 5. Conclusion

Dengue fever is a major health problem in Colombia due to its higher incidence and lethality in the younger adult population. In 2010, scholar population was especially susceptible to DENV infection in Medellin [9]. This study consisting of a serologic surveillance during two different epidemiological periods in schoolchildren population determined as “*sentinel population*” revealed high rates of DENV transmission. The significant increase of seroconversion and seroprevalence rates observed in schoolchildren raises the concern about a future increase in the severity of dengue cases in this population. Moreover, we showed how poor socioeconomic living conditions and densest human and mosquito populations significantly affected the dynamics of the dengue 2010 outbreak. Despite the fact that our analysis has limited factors (adult population, meteorological factors, vector competences, and human genetic background were not included in the analysis), our findings contribute to improve our knowledge about local burden of dengue virus and may allow to target population for effective prevention campaigns and vaccine introduction.

## Figures and Tables

**Table 1:** General characteristics of the studied schoolchildren population through 2010–2012, Medellin, Colombia.

	2010	2011	2012	Overall
	*n*=1,788	*n*=1,888	*n*=709	*n*=4,385
*Gender*	% (*n*)	% (*n*)	% (*n*)	% (*n*)
Male	48.5 (867)	47.2 (891)	47.1 (334)	47.7 (2,092)
Female	51.5 (921)	52.8 (997)	52.9 (375)	52.3 (2,293)

*Self-reported ethnic group*				
Mestizo	95.0 (1,698)	96.5 (1,800)	98.0 (695)	96.1 (4,193)
Afro-Colombian	5.0 (90)	3.5 (65)	2.0 (14)	3.9 (169)

*Age group (years)*				
5 to 9	31.9 (571)	21.1 (399)	26.5 (188)	26.4 (1,158)
10 to 14	54 (965)	58.1 (1,097)	58.4 (414)	56.5 (2,476)
15 to 19	14.1 (252)	20.8 (392)	15.1 (107)	17.1 (751)

*Age median (IQR)*	11 (9–13)	12 (10–14)	11 (9–13)	12 (9–14)

*Socioeconomic status*				
Low	56.9 (1,017)	60.3 (1,137)	48.6 (343)	57.0 (2,497)
Middle	36.2 (648)	34.5 (651)	51.4 (363)	38 (1,662)
High	6.9 (123)	5.1 (97)	0.0 (0)	5.0 (220)

IQR: interquartile range.

**Table 2:** Univariate logistic regression for DENV IgG seroprevalence in the studied schoolchildren population in 2010–2012 from Medellin.

	2010 (*n*=1,788)	Crude OR	95% CI	2011(*n*=1,888)	Crude OR	95% CI	2012 (*n*=709)	Crude OR	95% CI
*Gender*	% (*n*)			% (*n*)			% (*n*)		
Male	52.9 (459/867)	Ref.	—	57.2 (510/891)	Ref.	—	59.9 (200/334)	Ref.	—
Female	54.6 (503/921)	1.03	0.95–1.12	59.6 (594/997)	1.04	0.96–1.12	68.8 (258/375)	1.15^*∗*^	1.03–1.28

*Self-reported ethnic group*									
Afro-descent	45.6 (41/90)	Ref.	—	63.1 (41/65)	Ref.	—	71.4 (10/14)	Ref.	—
Mestizo	54.2 (921/1,698)	1.19	0.95–1.50	58.8 (1,058/1,800)	0.93	0.77–1.13	64.5 (448/695)	0.90	0.64–1.26

*Age group (years)*									
5 to 9	34.9 (199/571)	Ref.	—	45.4 (181/399)	Ref.	—	50 (94/188)	Ref.	—
10 to 14	61.6 (594/965)	2.99^*∗*^	2.41–3.71	60.2 (660/1,097)	1.90^*∗*^	1.51–2.40	70.5 (292/414)	2.40^*∗*^	1.68–3.41
15 to 19	67.1 (169/252)	3.80^*∗*^	2.78–5.21	67.1 (264/392)	2.60^*∗*^	1.95–3.47	64.6 (72/107)	2.06^*∗*^	1.25–3.40

*Socioeconomic status*									
Low	58.2 (592/1,017)	Ref.	—	60.0 (682/1,137)	Ref.	—	61.2 (210/343)	Ref.	—
Middle	49.1 (318/648)	0.69^*∗*^	0.57–0.84	56.5 (368/651)	0.87	0.72–1.6	68.0 (247/363)	1.37^*∗*^	1.0–1.85
High	42.3 (52/123)	0.53^*∗*^	0.36–0.77	54.6 (53/97)	0.80	0.53–1.22	0 (0/0)	—	—

*Study year*									
IgG positive	53.8 (962/1,788)	Ref.	—	58.8 (1,104/1,888)	1.21^*∗*^	1.06–1.38	64.6 (458/709)	1.57^*∗*^	1.30–1.87

^*∗*^Statistically significant: *p* < 0.05; OR: odds ratio; CI: confidential interval.

**Table 3:** Multivariate logistic regression for DENV IgG seroprevalence in the studied schoolchildren population in 2010–2012 from Medellin.

	2010			2011			2012		
	aOR	95% CI	*p* value	aOR	95% CI	*p* value	aOR	95% CI	*p* value
*Gender*									
Female	1.03	0.84–1.25	0.770	—	—	—	1.38	1.01–1.89	0.043

*Age group (years)*									
5 to 9	Ref.	—	—	Ref.	—	—	Ref.	—	—
10 to 14	2.96	2.38–3.68	<0.001	1.90	1.51–2.40	<0.001	2.32	1.62–3.31	<0.001
15 to 19	3.71	2.70–5.09	<0.001	2.59	1.94–3.46	<0.001	1.97	1.20–3.24	0.008

*Socioeconomic status*									
Low	Ref.	—	—	Ref.	—	—			
Middle	0.70	0.57–0.86	0.001	0.86	0.71–1.05	0.150			
High	0.59	0.39–0.87	0.009	0.91	0.59–1.39	0.663			

aOR: adjusted odds ratio.

**Table 4:** Characterization of antibody response to dengue virus serotypes using the ELISPOT-MNT_50_ in the studied schoolchildren population in 2010–2012 from Medellin.

	2010	2011	2012	Overall
	*n*=176	*n*=89	*n*=13	*n*=278
	% (*n*)	% (*n*)	% (*n*)	% (*n*)

*Negative*	2.8 (5)	7.9 (7)	7.7 (1)	4.7 (13)

*Monotypic*	26.7 (47)	19.1 (17)	0.0 (0)	23.0 (64)
DENV1	4.0 (7)	5.6 (5)	—	4.3 (12)
DENV2	6.2 (11)	5.6 (5)	—	5.6 (16)
DENV3	1.1 (2)	0.0 (0)	—	0.7 (2)
DENV4	15.3 (27)	7.9 (7)	—	12.2 (34)

*Multitypic*	70.4 (124)	73.0 (65)	92.3 (12)	72.3 (201)
DENV1/2	0.0 (0)	3.4 (3)	0.0 (0)	1.1 (3)
DENV1/3	1.1 (2)	0.0 (0)	0.0 (0)	0.7 (2)
DENV1/4	5.1 (9)	6.7 (6)	15.4 (2)	6.1 (17)
DENV2/3	1.7 (3)	0.0 (0)	0.0 (0)	1.1 (3)
DENV2/4	17.0 (30)	5.6 (5)	7.7 (1)	12.9 (36)
DENV3/4	2.8 (5)	0.0 (0)	0.0 (0)	1.8 (5)
DENV1/3/4	4.5 (8)	1.1 (1)	23.1 (3)	4.3 (12)
DENV2/3/4	2.3 (4)	0.0 (0)	0.0 (0)	1.4 (4)
DENV1/2/4	19.9 (35)	22.5(20)	7.7 (1)	20.1 (56)
DENV1/2/3/4	15.9 (28)	33.7 (30)	38.5 (5)	22.7 (63)

## Data Availability

The data used to support the findings of this study are available from the corresponding author upon request.
